# Microbiota and Diapause-Induced Neuroprotection Share a Dependency on Calcium But Differ in Their Effects on Mitochondrial Morphology

**DOI:** 10.1523/ENEURO.0424-22.2023

**Published:** 2023-07-21

**Authors:** Scarlett E. Delgado, Arles Urrutia, Florence Gutzwiller, Chiayu Q. Chiu, Andrea Calixto

**Affiliations:** Centro Interdisciplinario de Neurociencia de Valparaíso, Instituto de Neurociencia, Facultad de Ciencias, Universidad de Valparaíso, Valparaíso 2340000, Chile

**Keywords:** calcium, diapause, microbiota, mitochondria, neuroregeneration, touch receptor neurons

## Abstract

The balance between the degeneration and regeneration of damaged neurons depends on intrinsic and environmental variables. In nematodes, neuronal degeneration can be reversed by intestinal GABA and lactate-producing bacteria, or by hibernation driven by food deprivation. However, it is not known whether these neuroprotective interventions share common pathways to drive regenerative outcomes. Using a well established neuronal degeneration model in the touch circuit of the bacterivore nematode *Caenorhabditis elegans*, we investigate the mechanistic commonalities between neuroprotection offered by the gut microbiota and hunger-induced diapause. Using transcriptomics approaches coupled to reverse genetics, we identify genes that are necessary for neuroprotection conferred by the microbiota. Some of these genes establish links between the microbiota and calcium homeostasis, diapause entry, and neuronal function and development. We find that extracellular calcium as well as mitochondrial MCU-1 and reticular SCA-1 calcium transporters are needed for neuroprotection by bacteria and by diapause entry. While the benefits exerted by neuroprotective bacteria require mitochondrial function, the diet itself does not affect mitochondrial size. In contrast, diapause increases both the number and length of mitochondria. These results suggest that metabolically induced neuronal protection may occur via multiple mechanisms.

## Significance Statement

Calcium signaling and mitochondrial function have recently been suggested to promote axonal growth following neuronal damage, but the underlying mechanisms and physiological significance are unclear. Combining transcriptomics, genetics, and cell biological approaches in a simple animal model of axonal degeneration and regeneration, we demonstrate that neuronal repair conferred by two different metabolic processes occurs in diverse ways, requiring differential changes in mitochondrial function and calcium homeostasis. Furthermore, this work shows that neuroprotection can be additive, providing a new conceptual framework for developing therapeutic interventions in neurodegenerative conditions that leverage the intersection of metabolism, microbiota, and mitochondrial function.

## Introduction

Axonal degeneration underlies many neuropathies and neurodegenerative diseases (Coleman and Perry, 2002) that involve a controlled dismantling of neuronal morphologic extensions (Saxena and Caroni, 2007). This process in the nematode *Caenorhabditis elegans* can be counteracted by developmental arrest induced by food deprivation (Calixto et al., 2012; Caneo et al., 2019) as well as by exposure to specific intestinal microbiota (Urrutia et al., 2020). The mechanisms that promote neuronal protection in both of these conditions are unclear. Is neuroprotection achieved actively as neuronal repair or merely the result of diminished neurodegenerative processes? Moreover, it is unknown whether ingested microbiota and metabolic state act similarly in this context.

Given the pivotal role of gene expression in the link between environment and cellular phenotype (Davidson and Levin, 2005; van Kesteren et al., 2011; Chen et al., 2015; Lu et al., 2022), identifying transcriptional changes at various stages of recovery may hold the key to understanding novel mechanisms and pathways for neuroprotection.

The microbiota composition and health are intimately interconnected with diet (Scott et al., 2013; Portune et al., 2017; Illiano et al., 2020; Jackson and Theiss, 2020). Through this relationship, metabolites produced by the microbiota have been linked to the development of neurodegenerative disorders (Chandra et al., 2020; Goyal et al., 2021; Milošević et al., 2021; Zhang et al., 2022). Dysbiosis of the gut microbiota is associated with inflammation, reactive oxygen species (ROS) increment, and mitochondrial dysfunction (Yardeni et al., 2019; Jackson and Theiss, 2020).

Oxidative damage is central to neurodegeneration, and mitochondria are key to the neutralization of oxidative stress (Kim et al., 2015; Singh et al., 2019; Ballard and Towarnicki, 2020). The effects associated with failure in the neutralization of ROS include impairment of synthesis and transport of lipids, and Ca^2+^ transport (Vance, 2014). Loss of mitochondrial homeostasis has been linked to degenerative processes in different animal and cell models (Pathak et al., 2013; Błaszczyk, 2020). In mice, intracellular calcium transporters, such as the mitochondrial calcium uniporter, have been shown to contribute to degeneration (Calvo-Rodriguez et al., 2020; Calvo-Rodriguez and Bacskai, 2021). However, mitochondria maintain cellular homeostasis by also modulating energy production and capture of calcium (Beal, 1996; Shvartsman et al., 2007; Ferrer, 2009; Picard and McEwen, 2014). Calcium signaling in microdomains is important in determining whether neuronal regeneration or degeneration occurs (Xu et al., 2001; Berridge, 2006; Yan and Jin, 2012) and involves the participation of the endoplasmic reticulum (ER) and mitochondria.

To gain mechanistic insight on the commonalities between microbiota and diapause induced neuroregeneration, we use a genetically encoded insult to the touch circuit of *C. elegans* (Driscoll and Chalfie, 1991; Calixto et al., 2012), whereby a mutation triggers a loss of selectivity of the MEC-4 channel (Shi et al., 2018), leading to an increase in cytoplasmic calcium and the subsequent energetic collapse of the neuron (Driscoll and Chalfie, 1991; Xu et al., 2001; Bianchi et al., 2004; Shi et al., 2018). Using this model, we investigate here the contribution of gene expression, mitochondria morphology, and the need for calcium in these two interventions.

## Materials and Methods

### *C. elegans* growth and maintenance

Wild-type, transgenics, and mutant *C. elegans* were maintained at 20°C, as reported previously (Brenner, 1974). The following nematode strains were used: wild type (N2); TU2773 [*uIs31(Pmec-17mec-17::gfp); mec-4d(e1611)X*]; WCH6 [*uIs71[Pmec-18sid-1; Pmyo-2mcherry], uIs31[Pmec-17mec-17::gfp], sid-1[pk3321], mec-4d[e1611]*]; *js609 [jsIs609:Is[Pmec-4::MLS::gfp]*]; and WCH42 [*jsIs609:Is[Pmec-4::MLS::gfp]; mec-4d [e1611]*].

All animals were maintained in *Escherichia coli* OP50 before using or feeding with other bacteria.

### Bacterial growth

*E. coli* OP50 and *E. coli* HT115 bacteria were grown overnight on Luria-Bertani (LB) plates at 37°C from glycerol stocks kept at −80°C. The next morning, a large amount of the bacterial lawn was inoculated in LB broth and grown for 6 h with agitation at 200–220 rpm at 37°C. Three hundred microliters of bacterial culture was seeded onto 60 mm nematode growth media (NGM) plates and allowed to dry overnight.

### *C. elegans mec-4d* transcriptomic analysis

Total RNA was isolated from synchronized *C. elegans mec-4d* populations feeding on *E. coli* OP50, or *E. coli* HT115, at 12, 24, and 48 h after hatching using RNA-Solv Reagent (Omega Biotek). Spectrophotometric quantification and integrity of RNA were determined on a bioanalyzer (model 2100, Agilent Technologies). mRNA libraries were prepared with the TruSeq RNA Sample Prep Kit (Illumina) according to the manufacturer protocol. The quality and size distribution of the libraries were evaluated with the model 2100 Bioanalyzer using a DNA 1000 chip (Agilent Technologies) and were quantified using the KAPA Library Quantification Kit for Illumina Platforms (Kapa Biosystems), on the Step One Plus Real-Time PCR System (Applied Biosystems). The libraries were sequenced using the HiSeq paired-end protocol (2× 100 bp). High-quality reads were selected using Trimmomatic version 0.36 and mapped to the *C. elegans* reference genome (WormBase release ws235) using Tophat version 2.0.9. (Trapnell et al., 2012). The resulting bam files were transformed for visualization of mapping results in the University of California, Santa Cruz, genome browser (https://genome.ucsc.edu). The mapping results and HTSeq-count version 0.6.0 were applied with the intersection nonempty argument to get the more permissive count. Based on the counts, a differential expression (DE) analysis in R version 3.3.2 was performed, between conditions at the different time points using both DESeq2 (Love et al., 2014) and edgeR (Robinson et al., 2010). The threshold for DE analysis cutoffs was defined as log10|FC| > 1, and adjusted (adj) *p* value < 0.05. Data from results for the differentially expressed genes are displayed in heatmaps in Extended Data Figure 1-1, and data-mining scripts and processing is displayed in a repository in the following link: https://github.com/ArlesUrrutia/mec4dvsOPvsHT115. Results for adj *p* value and Log fold change (Log2FC) were cleaned for unmeasurable genes and then analyzed in Python version 3.9 for Volcano plot observations of both DE analyses (Extended Data Fig. 1-1). Data and data-mining results for the differentially expressed genes are displayed in Extended Data Table 1-2.

### Enrichment analysis

Gene ontology (GO) analysis was performed using the enrichment tool in WormBase (Angeles-Albores et al., 2016, 2018). GExplore (Hutter et al., 2009; Boeck et al., 2016) was used to find, among our set of RNA interference (RNAi)-positive genes, those that are highly expressed in the dauer stage.

### Cultures without calcium

To obtain calcium-free media, CaCl_2_ was not added to the NGM media. Bacteria used to feed animals was grown over a day with the addition of EGTA 500 mm (Winkler) in LB broth (Calixto et al., 2012).

### Dauer synchronization

After the detection of a few dauers by direct observation, plates were washed using 1% SDS, and the liquid containing mixed-stage animals was placed on an Eppendorf tube. The tube was centrifuged for 2 min at 2000 rpm, and the supernatant was removed. The pellet of nematodes was washed for 15 min with a solution of SDS 1% with 2,5 μg/ml carbenicillin (PhytoTechnology Laboratories) and 25 μg/ml amphotericin B (Fungizone, Thermo Fisher Scientific). Afterward, animals were washed three more times with sterile distilled water and antibiotics. The pellet was placed on NGM plates, and after 1 h the portion of agar where the pellet was placed was cut off using a stainless steel spatula then washed with the same mixture of sterile distilled water and antibiotics. Animals were centrifuged for 2 min at 4500 rpm and placed in cell culture plates (TrueLine) with distilled water and antibiotics, as mentioned before. The media were replaced every 2 weeks.

### RNA interference by feeding

Bacterial clones from Ahringer Library were taken from glycerol stocks and grown overnight on LB plates containing tetracycline (12.5 μg/ml; PhytoTechnology Laboratories). The next morning, a chunk of bacterial lawn was grown on liquid LB containing carbenicillin (50 μg/ml; PhytoTechnology Laboratories) for 8 h. NGM plates were prepared to add 1 mm IPTG (isopropyl β-d-1-thiogalactopyranoside), and 400 μl of bacterial growth was seeded. As a control for RNAi, *unc-22* dsRNA was used, which renders animals uncoordinated (Unc).

### RNAi in developing animals

Thirty to sixty newly hatched (0–2 h posthatching) L1s were placed on plates containing dsRNA expressing *E. coli* HT115 bacteria. Larvae and adults were removed with M9 from plates full of laid embryos. Embryos remained attached to the plates and were allowed to hatch for 2 h. L1 larvae were picked 0–2 h posthatching with a mouth pipette in M9 and placed on experimental NGM plates seeded with the desired bacterial clones. Silencing was tested on the TU2773 strain (systemic, non-neuronal RNAi), and WCH6 [touch receptor neuron (TRN)-specific RNAi; Calixto et al., 2010, 2012]. The morphology of the Anterior Lateral Microtubule (AVM) touch neuron was scored at 72 h posthatching.

### RNAi in dauers

To avoid contamination, 30–90 animals were seeded on five plates per RNA condition after hypochlorite treatment of gravid hermaphrodites. By direct observation, we detected the first day of dauers on plates. Nematodes were synchronized on day 2 or 3 depending on the number of dauers required for observation. We followed the protocol described above as dauer synchronization. After some animals crawled out of the drop, 25 individuals were picked for observation under the microscope.

### Scoring of neuronal integrity

For morphologic evaluation, worms were mounted on 2% agarose pads. Dauers were mounted and paralyzed with 20 mm levamisole, and developing animals with 1 mm levamisole. Morphologic categories were assigned using the same criteria as in the study by Urrutia et al. (2020). Neurons with full-length axons, as well as those with anterior processes that passed the point of bifurcation to the nerve ring, were classified as AxW. Axons with only a process connected to the nerve ring were classified as AxL, and those that did not reach the bifurcation to the nerve ring were classified as AxT. Lack of axon and soma only was classified as AxØ, and the total absence was indicated as AxØ-S. For simplicity, all graphs show only the AxW category.

### Microscopy and photography

Images were taken using a Remote Pro DSLR (Breeze Systems), a camera (Rebel T3i, Canon), and a fluorescence microscope (Eclipse Ni-U, Nikon). Configuration was set up at 1/10 exposure time and ISO correction at 200 for fluorescence images.

### Quantitative measurements of mitochondrial morphology

Thirty to sixty newly hatched *js609* or WCH42 animals were placed on plates seeded with *E. coli* OP50 or *E. coli* HT115, and images of the mitochondria of AVM axons of L2 animals were taken after 24 h. Dauers were obtained 1 week later from the same plate by 1% SDS treatment, placed on sterile NGM plates devoid from bacteria to allow dauers to crawl away from the 1% SDS drop. Living dauers were mounted on 2% agarose pads, and photographs were taken at different focal points to register all mitochondria in each AVM. Each set was measured separately using ImageJ, and each mitochondrion was registered independently. First, we used a Neubauer chamber to standardize the observed length in pixels from the image. Then, using the line tool from ImageJ, we measured the number of pixels that each mitochondrion had in the image. With a scale entered in ImageJ, we obtained the length in micrometers. Each value was recorded in Excel, and, from that, the average value and the number of mitochondria in each axon were calculated.

Mitochondria <2 μm were classified as fragmented, between 2 and 4 μm as intermediate, and >4 μm mitochondria as filamentous (Neve et al., 2020).

### Experimental design and statistical analyses

#### Functional validation by RNAi

The functional validation of the genes identified as upregulated was tested by feeding animals with dsRNA (RNAi). RNAi experiments used *unc-22* dsRNA as control, which produces twitching in nematodes. When the plate has >70% of Unc animals, the experiment is considered valid. Statistical analysis is performed using an *E. coli* HT115 that does not express dsRNA as a control (one-way ANOVA).

#### Calcium depletion

Calcium chelators such as EGTA can reduce the degeneration rate in *mec-4d* animals (Calixto et al., 2012). Animals cultured in absence of environmental calcium were compared in a two-way ANOVA test to animals grown in NGM (CaCl_2_, 1 mm) at 72 h after hatching, given the same bacterial diet. Similar comparisons are performed for 1- to 2-week-old dauers in the absence of calcium using the same type of test. The role of intracellular calcium transporters like *sca-1* and *mcu-1* in neuroprotection was tested using RNAi and compared with an *E. coli* HT115 control for developing (72 h posthatching) and dauer animals. For developing animals, one-way ANOVA was used, and for dauers, a two-way ANOVA was used.

#### Mitochondrial measurements

Analysis of mitochondrial number and length was performed using one-way ANOVA comparisons in wild-type and *mec-4d* genetic backgrounds. Specific comparisons included evaluating animals at the same developmental stage (L2 or dauer) in different bacterial diets and comparing L2 with dauers within the same diet. For the functional testing of mitochondrial genes, RNAi was performed in TRN-specific and systemic strains carrying the *mec-4d* mutation and the scoring was performed at 72 h posthatching. The *E. coli* HT115 diet served as the control for each gene tested. One-way ANOVA was used to assess both the treatment and control groups. Pearson’s correlation analysis was used to examine the relationship between mitochondrial number and length with the percentage of wild-type axons (AxW) observed in *mec-4d* animals L2 and dauers.

All experiments were performed at least three times (three biological replicas, started on different days and from different parental nematodes). Each biological replica contained a triplicate (three technical replicas). Statistical evaluation was performed by a one-way or two-way ANOVA with *post hoc* analyses.

Criteria for data exclusion were as follows: we excluded experimental replicas when there was contamination with unwanted bacteria or fungi on the nematode plates or when bacteria had been almost or completely consumed.

## Results

### Transcriptomic profiling of diet induced neuroprotection in developing *C. elegans*

**Figure 1.**
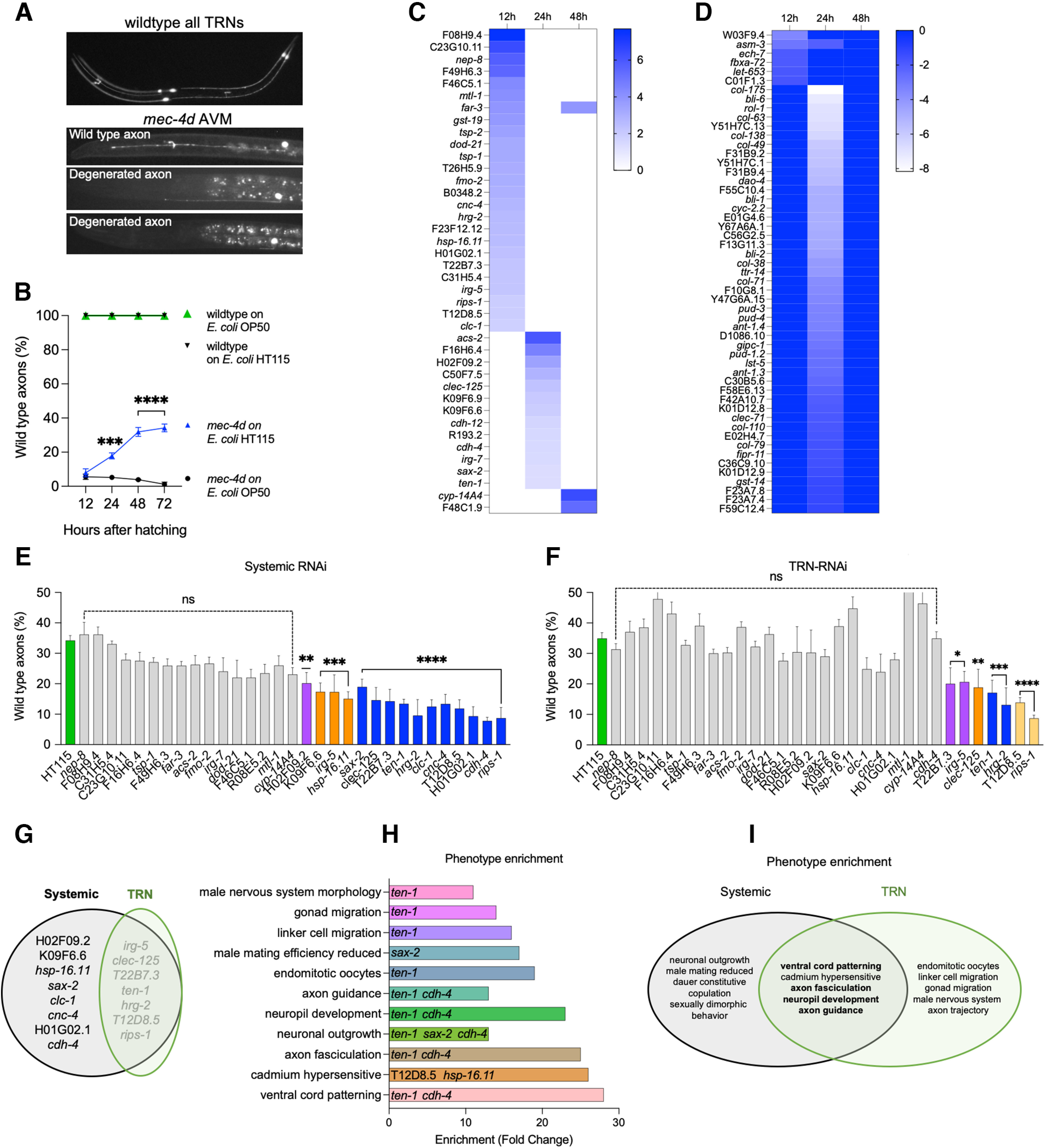
Gene expression analysis of microbiota induced neuronal protection and *in vivo* validation of neuroprotective gene candidates. ***A***, Top, Photography of a wild-type nematode expressing gfp in all the TRNs. Bottom panels, *mec-4d* animals expressing gfp in the AVM with different degrees of protection/degeneration. Wild-type axons (AxW) are considered the protected category. ***B***, Time course of wild-type axonal morphology (AxW) in wild-type and *mec-4d* animals grown on *E. coli* OP50 and *E. coli* HT115 diets at 12, 24, 48, and 72 h posthatching (*N* = 3; two-way ANOVA). ***C***, ***D***, Heatmaps of upregulated (***C***) and downregulated (***D***) genes in *E. coli* HT115 diet compared with *E. coli* OP50 at 12, 24, and 48 h posthatching. Darker blue indicates higher levels of expression. ***E***, ***F***, Percentage of AxW morphology in animals feeding on dsRNA-expressing bacteria of upregulated genes in a systemic strain (***E***) and a TRN-specific strain (***F***), 72 h post-hatching (one-way ANOVA, N = 3 or more). ***G***, Venn diagram showing the common and unique genes between those functionally required for *E. coli* HT115 protection systemically and TRNs specifically. ***H***, Gene enrichment for the phenotype of all genes required for neuroprotection. ***I***, Venn diagram showing the common and unique phenotypical enrichment categories of genes required systemically and TRN specifically. 0.1234(ns), 0.0332(*), 0.0021(**), 0.0002(***), 0.0001(****). Heatmaps and Volcano plots of transcriptomics analysis can be found in Extended Data Figure 1-1. Additional enrichment analyses are shown in Extended Data Figures 1-2, 1-3, and 1-4. Differentially expressed genes are listed in Extended Data Table 1-1. All genes used for enrichment analysis, and their statistics are shown in Extended Data Tables 1-2 and 1-3.

Intestinal bacteria and their metabolites can define the degeneration rate of TRNs of developing *C. elegans* expressing the *mec-4d* degenerin (Fig. 1*A*; Urrutia et al., 2020). Specifically, GABA and lactate-producing *E. coli* HT115 are protective in contrast with *E. coli* OP50, which does not produce the metabolites (Fig. 1*B*). To identify the nematode genes that may underlie diet-induced neuroprotection, we performed RNAseq of *mec-4d* animals feeding on *E. coli* HT115 and *E. coli* OP50 at 12, 24, and 48 h posthatching. Differential expression analysis was performed comparing animals fed on *E. coli* HT115 with those fed on *E. coli* OP50 at each time point. Ninety-three genes were differentially expressed, most of which were found in earlier development (12 and 24 h; Extended Data Fig. 1-1, Extended Data Table 1-1). Forty genes were upregulated in animals fed *E. coli* HT115 (Fig. 1*C*), while 53 were downregulated (Fig. 1*D*). Upregulated genes were phenotypically enriched (Angeles-Albores et al., 2016) in neuronal development, axonal pathfinding, and axonal outgrowth, as well as in diapause formation (Extended Data Fig. 1-2), and were enriched in the GO terms calcium binding, neuronal development, the unfolded protein response, and biotic interactions, among other (Extended Data Fig. 1-2, Extended Data Table 1-2). This shows that the *E. coli* HT115 diet increases expression of genes involved in neuronal processes that can be detected even when the pool of RNAs used for the analysis is from the whole nematode and not a neuron-specific transcriptome. The comparison of our results with previous published data showed little overlap, probably because of differences in the nematode genotype and the developmental stage of RNA collection in adult wild-type (MacNeil et al., 2013) and adult *glp-4* mutants (Revtovich et al., 2019) in contrast with our analysis performed in *mec-4d* at three different larval stages (L1, L2-L3, and L4). Moreover, GO and phenotype enrichment of previous data did not contain any neuronally enriched functions (Extended Data Table 1-3), suggesting that the protective diet increased the expression of ad hoc genes required for repair in *mec-4d* background. On the other hand, downregulated genes in *E. coli* HT115 were associated with body morphology phenotypes (Extended Data Fig. 1-2) and with GO terms such as ATP synthesis and other functions that reside in the mitochondria, in addition to cuticle development (Extended Data Fig. 1-2). Interestingly, previous transcriptomics coincide with enrichment in mitochondrial processes, although the genes are not shared (MacNeil et al., 2013; Revtovich et al., 2019), suggesting that *E. coli* HT115 has a role over mitochondrial function regardless of genetic background.

10.1523/ENEURO.0424-22.2023.f1-1 Figure 1-1 ***A***, Heatmap of genes DE in *E. coli* HT115 compared with *E. coli* OP50 at different times during development. DeSeq and EdgeR analyses are shown. ***B***, Volcano plot for DE analysis on each developmental timepoint. Download Figure 1-1, TIF file.

**Figure 2.**
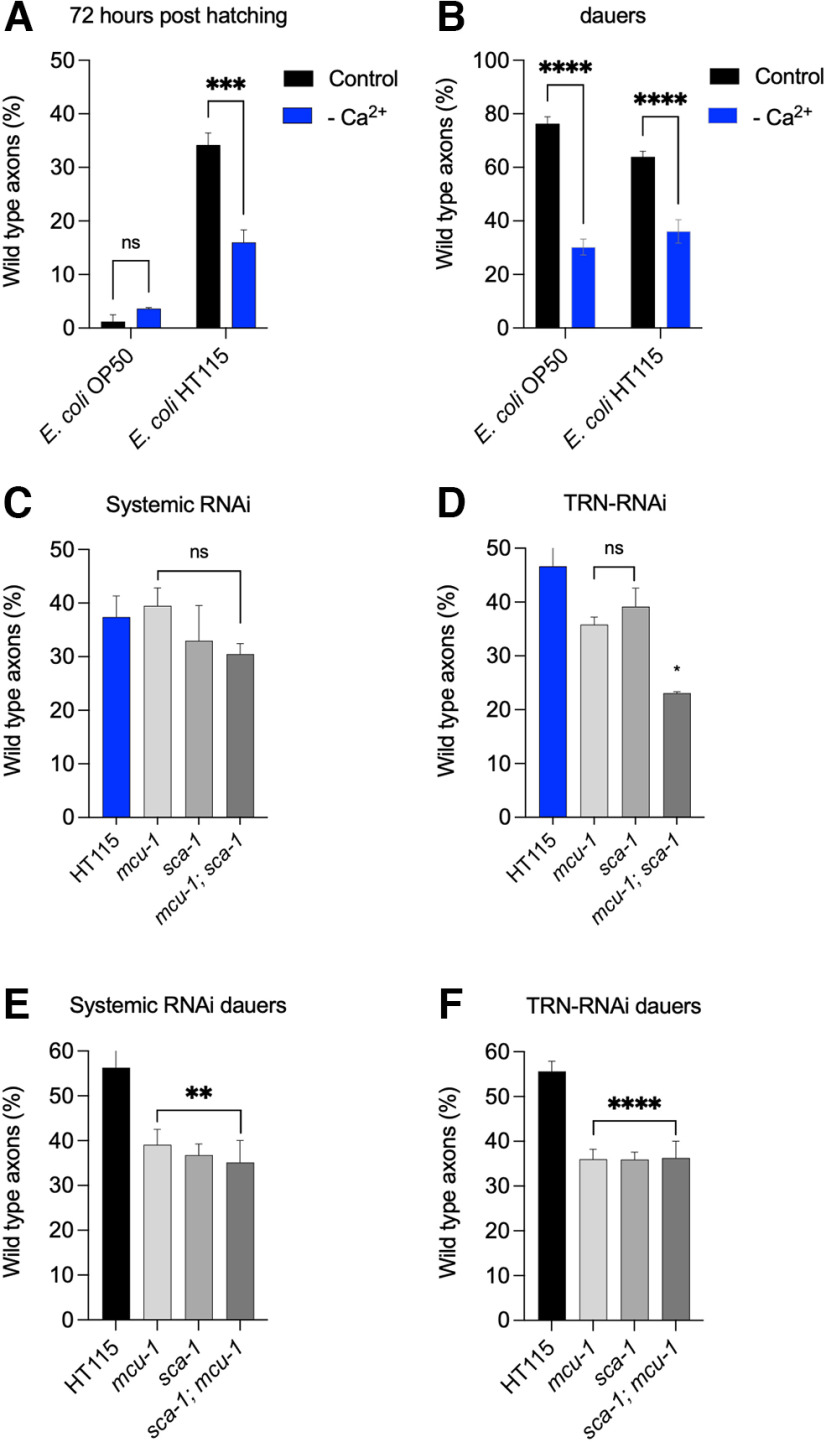
Mitochondrial and ER calcium transporters are required TRN autonomously for neuroprotection induced by diet-induced and diapause-induced regeneration. ***A***, Percentage of AxW morphology in *mec-4d* animals at 72 h posthatching in *E. coli* OP50 and *E. coli* HT115 with and without calcium (*N* = 3; two-way ANOVA). ***B***, Percentage of AxW morphology in dauer *mec-4d* animals cultured before synchronization in *E. coli* OP50 and *E. coli* HT115 with or without calcium (*N* = 3; two-way ANOVA). ***C***, ***D***, Percentage of AxW morphology in *mec-4d* animals on *sca-1* and *mcu-1* dsRNA-expressing bacteria 72 h posthatching in systemic strains (***C***) and TRN RNAi-specific strains (***D***; *N* = 4; one-way ANOVA). ***E***, ***F***, Percentage of AxW morphology in *mec-4d* animals feeding on *sca-1* and *mcu-1* dsRNA-expressing bacteria in dauers in systemic strains (***E***) and TRN-specific strains (***F***; *N* = 4; one-way ANOVA). 0.1234(ns), 0.0332(*), 0.0021(**), 0.0002(***), 0.0001(****).

10.1523/ENEURO.0424-22.2023.f1-2 Figure 1-2 Enrichment analysis of genes differentially expressed in *E. coli* HT115 diet. ***A–D***, Phenotype (***A***, ***C***) and gene ontology (***B***, ***D***) enrichment of genes upregulated (***A***, ***B***) and downregulated (***C***, ***D***) in *E. coli* HT115 compared with *E. coli* OP50. Statistical analyses are shown in Extended Data Table 1-3. Download Figure 1-2, TIF file.

**Figure 3.**
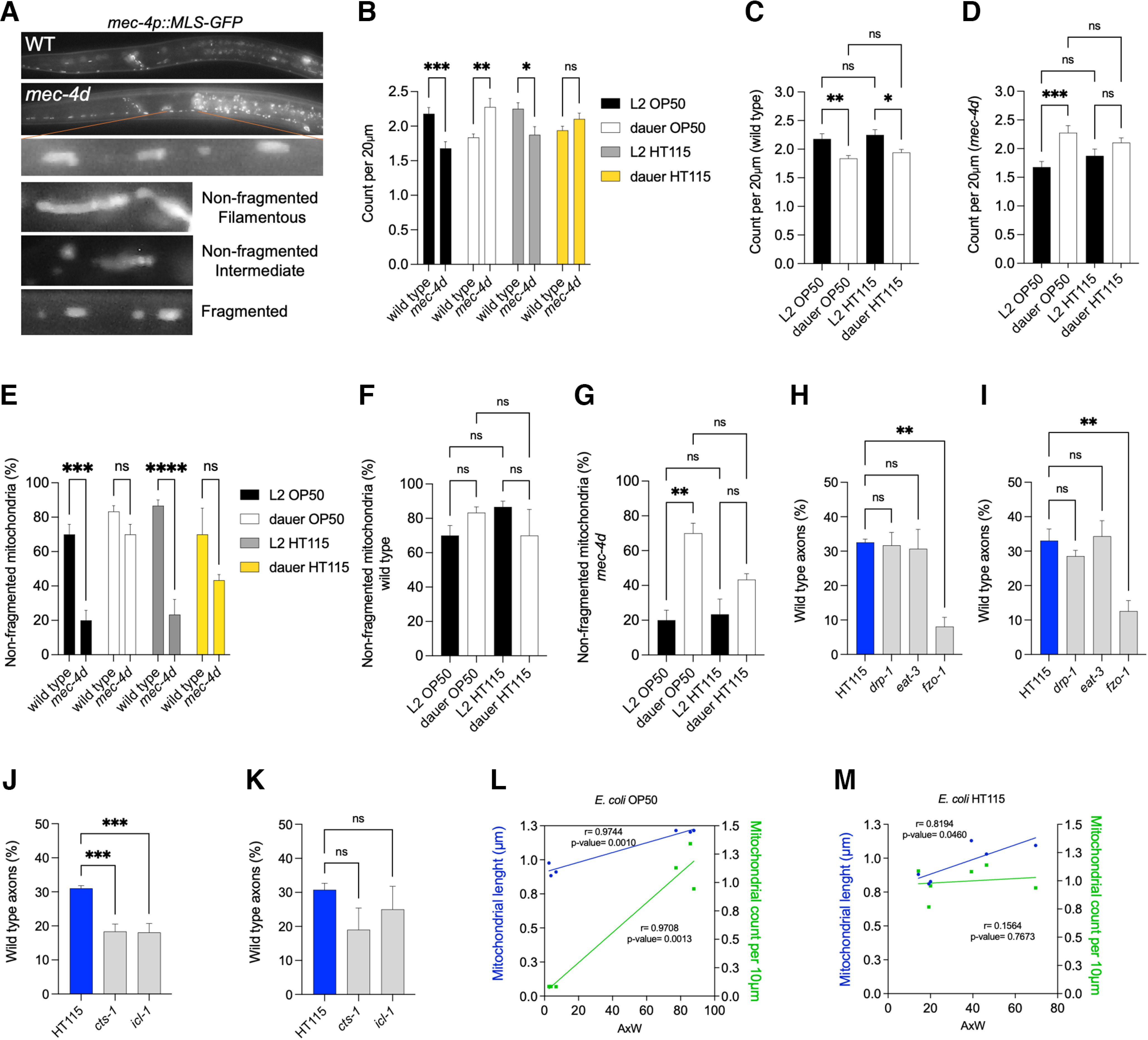
Diapause improves mitochondrial defects caused by a degenerin mutation in the TRNs. ***A***, Representative images of wild-type or *mec-4d* dauers previously fed on *E. coli* OP50 expressing *mec-4p::MLS-GFP*. Inset, Mitochondria of different sizes in a section of the AVM axon. Bottom panels, Representative images of filamentous, intermediate, and fragmented mitochondria expressing mitochondrial *gfp.* Filamentous and intermediate mitochondria are referred to as nonfragmented. ***B–D***, Number of mitochondria normalized by axonal length in TRNs growing in *E. coli* OP50 and *E. coli* HT115 in wild-type (***B***, ***C***) and *mec-4d* (***B***, ***D***) strains in L2 and early dauer stages (*N* = 3; one-way ANOVA). ***E–G***, Percentage of the population that exhibits nonfragmented mitochondria in TRNs wild-type animals (***E***, ***F***) and *mec-4d* animals (***E***, ***G***) growing *E. coli* OP50 or *E. coli* HT115 in L2 and early dauers (*N* = 3; one-way ANOVA). ***H–K***, Percentage of AxW morphology in animals feeding on dsRNA-expressing bacteria of mitochondrial genes in a TRN-specific (***H***, ***J***) or systemic-specific (***I***, ***K***) strain 72 h posthatching (*N* = 3 or 4; one-way ANOVA). 0.1234(ns), 0.0332(*), 0.0021(**), 0.0002(***), 0.0001(****). ***L***, ***M***, Correlation between mitochondrial length and the number of mitochondria normalized by the length in *mec-4d* animals growing in *E. coli* OP50 (***L***) and *E. coli* HT115 (***M***) in L2 and dauers.

10.1523/ENEURO.0424-22.2023.f1-3 Figure 1-3 Enrichment analysis of genes required for neuroprotection conferred by *E. coli* HT115. ***A***, Gene ontology of genes that are required for *E. coli* HT115 neuroprotection. ***B***, Venn diagram of Gene ontology categories of genes required systemically and in the TRNs for neuroprotection. Statistical analyses are shown in Extended Data Table 1-3. Download Figure 1-3, TIF file.

10.1523/ENEURO.0424-22.2023.f1-4 Figure 1-4 ***A***, ***B***, Enrichment analysis of genes upregulated in dauers that are shared with genes required for neuroprotection in *E. coli* HT115. ***A***, ***B***, Gene ontology enrichment associated with genes shared between *E. coli* HT115 RNAi-positive clones and dauers (***A***), and those not shared with dauers (***B***). Statistical analyses are shown in Extended Data Table 1-3. Download Figure 1-4, TIF file.

We tested whether genes that increase their expression in *mec-4d* fed on *E. coli* HT115 contribute to neuroprotection *in vivo*. This can be functionally tested by targeted silencing using RNAi interference (Fraser et al., 2000; Kamath et al., 2003). We used *E. coli* HT115 expressing dsRNA for 31 of the 40 upregulated genes to study their contribution to neuroprotection in *mec-4d* animals. The remaining nine clones were not available in the feeding RNAi library or caused embryonic lethality (Fraser et al., 2000; Kamath et al., 2003). To dissect whether the function of these genes is required in the touch neurons or systemically, we used strains with TRN-specific and systemic RNAi (Calixto et al., 2010). Fifteen dsRNAs caused a decrease in neuroprotection when silenced in non-neuronal tissues (Fig. 1*E*,*G*). Of those, seven were needed for protection specifically in the TRNs (Fig. 1*F*,*G*). A large proportion of genes necessary for neuroprotection in *mec-4d* animals are neuronally expressed, including the TRNs (Table 1; Taylor et al., 2021), and show functional clustering (Angeles-Albores et al., 2016) in neuronal phenotypes (Fig. 1*H*). We compared the functional enrichment provided by genes only required systemically with those required in the TRNs and found a large overlap in neuronal phenotypes despite having come from different gene pools (Fig. 1*I*). Clustering by GO of RNAi-positive genes also showed enrichment in neuronal categories (Extended Data Fig. 1-3). GO enrichment showed TRN-specific axon guidance and axon projection terms, while system-specific categories were enriched in stress responses to biotic stimulus and to incorrectly folded proteins. The systemic and TRN enrichment in GO terms overlapped in neuronal development and immune defenses to microbes (Extended Data Fig. 1-3). This is coherent with a systemic contribution to lowering cellular stress while neuronal autonomous processes are clustered in categories related to neuronal repair.

**Table 1:** Genes confirmed by RNAi to be required for HT115-induced neuroprotection

Gene	TRN	Systemic	Function	Expression
*hrg-2*	***	****	Heme-binding activity	TRN, hypodermis
T12D8.5	****	****		TRN, sensory neurons, interneurons and intestine
*ten-1*	***	****	Neuronal and epidermal development	TRN, body muscle cell, gonad, hypodermis, interneurons and sensory neurons
T22B7.3	*	****		Sensory neurons, germline precursors, and hypodermis
*irg-5*	*	***	Defense response to Gram^+^ bacterium	Motor neurons, pharynx and intestine
*rips-1*	****	****	SAM-dependent methyltransferase	Sensory neurons, interneurons, motor neurons phanyngeal interneurons and intestine
*clec-125*	**	****	Carbohydrate binding activity	ASG neuron, germline
*hsp-16.11*		***	Involved in endoplasmic reticulum UPR	TRN, and all neurons
*cdh-4*		****	Axon guidance in the ventral cord	TRN, TRN and most neurons, muscle, and rectal gland cell
*sax-2*		****	Neuron projection development	TRN, and most neurons
*cnc-4*		****	Involved in defense response	Hypodermis, intestine, and seam cell
H01G02.1		****		GABAergic and dopaminergic neurons, intestine
H02F09.2		**		
K09F6.6		***		Motor neurons and intestine
*clc-1*		****	Defense response to Gram^+^ bacterium; epithelial cell–cell adhesion; and innate immune response	Pharyngeal interneurons and pharyngeal motor neurons
*cts-1*	***		Citrate synthase activity (TCA)	TRN, and most neurons, intestine, hypodermis, body wall muscle
*icl-1*	***		Isocitrate lyase activity	Intestine, hypodermis, muscle, pharynx

SAM, *S*-Adenosylmethionine; TCA, tricarboxylic acid. The *p* values indicate when the gene is required systemically and/or TRN autonomously for neuroprotection.

****p < 0.0001, *** < 0.001, **p < 0.005, *p < 0.05.

10.1523/ENEURO.0424-22.2023.tab1-1 Table 1-1 Genes differentially expressed in animals feeding on *E. coli* HT115 compared with *E. coli* OP50. The number of reads per replica are shown for each condition as well as the differential expression analysis performed using DeSeq and EdgeR. Download Table 1-1, XLS file.

10.1523/ENEURO.0424-22.2023.tab1-2 Table 1-2 Lists, enrichment analyses, and statistics of genes differentially expressed in *mec-4d* animals feeding on *E. coli* HT115 compared with *E. coli* OP50. Download Table 1-2, XLS file.

10.1523/ENEURO.0424-22.2023.tab1-3 Table 1-3 Lists, enrichment analyses, and statistics of genes overexpressed in animals feeding on *E. coli* HT115 compared with *E. coli* OP50 in previous works by others (MacNeil et al., 2013; Revtovich et al., 2019). Download Table 1-3, XLS file.

Diapause entry is also strongly neuroprotective (Caneo et al., 2019). We compared the transcriptomic results shown above to available data on dauer gene expression obtained by others (Hutter et al., 2009; Boeck et al., 2016). Four of the fifteen genes required for neuroprotection are also upregulated in dauers *sax-2*, *hsp-16.11*, *T22B7.3*, and *T12D8.5* (Boeck et al., 2016), suggesting that there is a common neuroprotective gene pool. These genes cluster in categories mainly related to neurogenesis and the unfolded protein response, probably to regulate cellular homeostasis after the early stress response (Extended Data Fig. 1-4). Genes that are not shared with dauers include categories mostly related to axonal guidance, development and body morphology development, and interspecies communication (Extended Data Fig. 1-4), suggesting that there are functional distinction between the two gene pools.

### Calcium depletion and neuroprotection

Transcriptomics analysis (Fig. 1) revealed that several genes related to calcium homeostasis are differentially expressed in neuroprotective conditions. This prompted us to look further into the calcium contribution to neuroprotection induced by diet and by diapause. *E. coli* HT115-fed *mec-4d* nematodes growing in media lacking calcium showed a significantly lower percentage of wild-type TRN axons (AxW) at 72 h after hatching (Fig. 2*A*), suggesting that calcium also contributes to axonal regrowth. In contrast, AxW counts in animals on the standard *E. coli* OP50 diet did not differ in low calcium. We next asked whether calcium plays a similar role in diapause-induced regeneration. *E. coli* OP50 and HT115-fed worms were induced to enter diapause by bacterial food exhaustion. Calcium removal in both dauer populations caused a significant decrease in wild-type axons (Fig. 2*B*). Notably, the extent of axonal regrowth and reduction in control and calcium-depleted conditions, respectively, did not differ between the two groups of dauers, suggesting that feeding on *E. coli* HT115 did not mask or attenuate diapause benefits. Together, the results support the idea that, although both microbiota-induced and diapause-induced neuroprotective processes require calcium, the underlying mechanisms are not completely overlapping.

### Mitochondrial and reticular calcium transporters in TRNs are required for neuroprotection

Given the observed dependency on calcium, we next asked whether calcium redistribution across different subcellular compartments by specific transporters plays a role in neuroprotection. For the *E. coli* HT115 diet during development and in diapause, we evaluated the requirement for *mcu-1*, the mitochondrial calcium uniporter, and *sca-1*, the sarco endoplasmic reticulum calcium ATPase (SERCA; Betzer et al., 2018; Csordás et al., 2018; Sarasija et al., 2018; Wang et al., 2019; Calvo-Rodriguez et al., 2020). These two transporters were silenced by feeding *mcu-1* and *sca-1* dsRNA-expressing bacteria to synchronized L1 *mec-4d* worms, and we scored neuronal integrity of the AVM TRN either 72 h later for developing animals (Urrutia et al., 2020) or after the second generation had become dauers (see detailed protocol in Materials and Methods).

At 72 h post-dsRNA treatment during development, only the simultaneous silencing of *mcu-1* and *sca-1* caused a reduction in AxW in the TRN-specific RNAi strain (Fig. 2*C*,*D*), suggesting that either transporter is sufficient for diet-induced neuroprotection. In dauers, on the other hand, silencing either *mcu-1* or *sca-1* impaired axonal regeneration with TRN-specific or systemic RNAi (Fig. 2*E*,*F*). Together, these results suggest that either calcium transporter can compensate for the loss of the other in *E. coli* HT115-mediated neuroprotection, whereas both are necessary systemically in dauer neuroprotection.

### Diapause induces increase in mitochondrial number and size in *mec-4d* animals

Regeneration under a chronic degenerative stimulus can be energetically demanding (Caneo et al., 2019). Specifically, MEC-4d overactivity imposes an ionic imbalance that can only be repaired by active Na^+^ extrusion such as would occur through the NaK ATPase (Davis et al., 1995; Calixto, 2015). Because mitochondria are central to cellular energetic control and have a core role in neuronal cell death (Dawson and Dawson, 2017) and in axonal degeneration in the *mec-4d* model (Calixto et al., 2012), we examined whether diet-induced and diapause-induced neuroprotection were associated with mitochondrial changes in TRNs. We measured mitochondrial number and length in the AVM neuron of animals expressing a fluorescent mitochondrial marker (*jsIs609:Is[Pmec-4::MLS::gfp]*) in both wild-type (Fatouros et al., 2012) and *mec-4d* backgrounds (Fig. 3*A*). These quantifications were performed in 1- or 2-week-old dauers and in L2 control animals because dauers have an L2d lineage (Karp and Ambros, 2012). Initially, we measured each mitochondrion and recorded each value separated by sample, then we examined mitochondrial values and categorized them into three distinct groups based on their size: filamentous, intermediate, and fragmented (Neve et al., 2020; Fig. 3).

First, we noted that *mec-4d* L2 animals have significantly less mitochondria in the TRNs than wild types in either diet (Fig. 3*B*). Second, regardless of genotype, mitochondria numbers are similar between diets (Fig. 3*C*,*D*). These two observations indicate that *E. coli* HT115 does not exert its neuroprotective effects through rescuing mitochondria reductions. However, *mec-4d* dauers grown on *E. coli* OP50, but not on *E. coli* HT115, have significantly more mitochondria than wild-type animals (Fig. 3*B*). Third, TRNs in wild-type dauers possess fewer mitochondria compared with their L2 controls (Fig. 3*C*), supporting the idea that diapause has lower metabolic demands under nondegenerative conditions. This decrease was observed regardless of diet in wild types but varied in *mec-4d* dauers. TRNs in mutant dauers previously fed with *E. coli* OP50, but not *E. coli* HT115, have significantly more mitochondria than their respective wild-type dauers (Fig. 3*B*) and L2 mutants (Fig. 3*D*), suggesting that mitochondria increase may be a mechanism in dauers to alleviate homeostatic stress in *mec-4d* TRNs in a nonprotective diet.

Nonfragmented mitochondria are considered to be optimized for function and may protect against neuronal damage (Chen et al., 2007; Kiryu-Seo and Kiyama, 2019; Wang et al., 2021). We wondered whether they play a role in diet-induced or diapause-induced neuroprotection. Each mitochondrion in the AVM axons was classified as nonfragmented (filamentous, intermediate) or fragmented. We represented the percentage of animals with the largest mitochondria (nonfragmented) in each condition (Fig. 3*E–G*). First, TRNs in L2 *mec-4d* mutants have smaller mitochondria than wild-type TRNs in either diet, suggesting that diet does not compensate for mitochondrial size changes that are associated with *mec-4d* in developing animals (Fig. 3*E*). In dauers, however, the number of nonfragmented mitochondria is similar between genotypes (Fig. 3*E*). Wild-type L2 and dauers have equal numbers of nonfragmented mitochondria, independent of the diet, suggesting that, in the absence of a prodegenerative stimulus, TRN mitochondria do not change in size (Fig. 3*F*). Importantly, *mec-4d* dauers have larger mitochondria than *mec-4d* L2 controls on *E. coli* OP50 (Fig. 3*G*), and there is a similar trend on *E. coli* HT115 (adj *p*-value, 0.06), supporting the idea that mitochondrial enlargement may account for diapause-mediated regeneration. We further evaluated the relationship between the percentage of wild-type axons observed and mitochondrial parameters. Linear regression analysis revealed a strong relationship between AxW morphology and mitochondrial length for animals on both diets (Fig. 3*H*,*I*), although no clear relationship between mitochondrial numbers and axonal regeneration can be stated on *E coli* HT115-fed worms (Fig. 3*I*). Thus far, our results support the idea that diapause-induced neuroprotection, but not diet-induced neuroprotection, is associated with mitochondrial changes in size and number.

To confirm the lack of association between diet and mitochondrial number and size, we further investigated whether HT115-induced protection requires genes involved in mitochondrial fusion (*eat-3*, *fzo-1*; Kanazawa et al., 2008)–mitochondrial fission (*drp-1*; Lu et al., 2011; Navarro-González et al., 2017; Byrne et al., 2019) dynamics using TRN-specific and systemic RNAi strains as before. Consistent with our previous evaluations, there was no significant effect on neuronal protection for *drp-1* or *eat-3* dsRNA (Fig. 3*G*,*H*). However, the silencing of *fzo-1*, known to be important for external membrane fusion of mitochondria, significantly decreased the number of wild-type axons. Interestingly, *fzo-1* is the ortholog of *mitofusins*, which in addition to mitochondrial fusion, stabilize the connection between mitochondria and ER, to, for example, transport calcium from the ER to the mitochondria (Decuypere et al., 2011; Michel and Kornmann, 2012; Herrera-Cruz and Simmen, 2017; Rodríguez-Arribas et al., 2017). Thus, *fzo-1* loss could be affecting axonal regrowth through these processes.

We tested the relevance of two other genes involved in mitochondrial metabolism, *cts-1* (citrate synthase, tricarboxylic acid cycle) and *icl-1* (isocitrate lyase, glyoxylate cycle), in axonal regrowth. Both of them are required for neuroprotection induced by diet in the TRN-specific RNAi strain (Fig. 3*G*,*H*), showing that metabolic function of mitochondria is critical for dietary neuronal protection in a cell-autonomous manner.

Together, our results suggest that diet-induced neuroprotection requires mitochondrial function in metabolism while diapause-induced neuroprotection may increase mitochondrial number and size.

## Discussion

Food availability and microbiota composition affect neuronal integrity and survival (Caneo et al., 2019; Liu et al., 2020; Urrutia et al., 2020; Shandilya et al., 2022; Zhang et al., 2022; Urquiza-Zurich et al., 2023). To find commonalities between these two neuroprotective conditions, we study gene expression, calcium contribution, and mitochondrial parameters in the AVM neuron of *C. elegans*. Transcriptomics analysis reveals that feeding on neuroprotective bacteria induces the expression of genes required for calcium dynamics, neurogenesis and neuronal function, and dauer formation. Removing extracellular calcium affects both diet-induced and diapause-induced neuroprotection. Interestingly, simultaneous silencing of both *mcu-1* and *sca-1* is necessary to prevent axonal regrowth induced by diet, whereas perturbation of either calcium transporter is sufficient to disrupt diapause-conferred neuroprotection. Moreover, larger mitochondria in the TRNs are promoted by diapause but not by protective microbiota.

### A neuroprotective gene pool?

The TRNs are highly regenerative cells capable of regrowth after axotomy (Wu et al., 2007) or under protective treatment in chronic models of damage such as the *mec-4d* degenerin (Caneo et al., 2019; Urrutia et al., 2020). Microbiota protection occurs early in development and is long-lasting (Urrutia et al., 2020), which is coherent with the early expression of neuroprotective genes found here. But how does the expression of these genes contribute to creating a protective environment and promote neuronal regrowth? We hypothesize that there are two components working in parallel, one systemic and one in the TRNs. Based on our transcriptomics analysis, we think that the systemic component includes direct effects of bacteria in the intestine by the secretion of specific metabolites (MacNeil et al., 2013), and bidirectional communication through immune genes such as *clc-1*, *irg-5*, and *cnc-4*. It also includes the expression of genes that operate outside the TRNs to create a propitious environment for regeneration (*crh-2*, *sax-2*), lowering redox stress and alleviating the energetic demand of repairing tissues (*hsp-16.11*). The cell-autonomous effect relies on TRN expression of genes that promote neuronal growth *in situ* on favorable conditions in the extracellular milieu (*ten-1*, *hrg-2*). Metabolites produced by *E. coli* HT115, such as GABA and lactate, could improve the stress status of the intestine of nematodes previously reared on *E. coli* OP50. For example, GABA production and extrusion through the GABA shunt (Feehily et al., 2013) is a mechanism used by bacteria to lower the acidic stress of the intestine of the host where they colonize. *E. coli* HT115, unlike *E. coli* OP50, contains all enzymes required for GABA production and export (Urrutia et al., 2020). An interesting class of genes that appeared in our screen and others (MacNeil et al., 2013; Revtovich et al., 2019) are the *hrg* (heme-responsive genes). Free heme is highly reactive and can intercalate in lipid bilayers (Chen et al., 2012). HRG-2, a heme deficiency-responsive membrane protein, regulates heme homeostasis and detoxification (Chen et al., 2012), alleviating cellular redox stress. This function is required for *E. coli* HT115-induced neuroprotection systemically and in the TRNs.

In parallel, *E. coli* HT115 metabolites could directly travel to the TRNs using specific transporters like UNC-47 or SNF-5; or could trigger signaling cascades such as those initiated by the transcription factor DAF-16/FOXO or through specific GABA receptors like GAB-1 or LGC-37, all of which are necessary for *E. coli* HT115 protection (Urrutia et al., 2020). Others studies have shown that *E. coli* HT115 lowers cellular stress in *C. elegans* by counteracting vitamin B12 deficiency and the toxic accumulation of propionate, most of which improves mitochondrial health (Revtovich et al., 2019). Bacterial metabolites and the induction of a nematode gene pool could then directly lower systemic mitochondrial stress (*hsp-16.11*) and promote specific functions in adjacent tissues to the TRNs. For example, adhesion molecules such as cadherins mediate cell signaling and neuroregeneration (Hirota et al., 2001; Kanemaru et al., 2013; Friedman et al., 2015; Yulis et al., 2018; Punovuori et al., 2021). The teneurins are transmembrane proteins fundamental for the development of the nervous system (Drabikowski et al., 2005; Mörck et al., 2010; Tucker et al., 2012; Topf and Drabikowski, 2019) and also are neuroprotective (Al Chawaf et al., 2007; Trubiani et al., 2007; Tessarin et al., 2019).

A few genes necessary for *E. coli* HT115-induced protection are also upregulated in dauers (Hutter et al., 2009; Boeck et al., 2016), suggesting that both signaling processes might share a common gene pool. Previous analysis shows that *C. elegans* diapause induces large transcriptional changes to accommodate periods of long-lasting starvation (Dalley and Golomb, 1992; Jones et al., 2001; Wang and Kim, 2003). Some of these changes include the overexpression of proregenerative genes such as *dlk-1* and the repression of antiregenerative genes such as *efa-6* (Chen et al., 2011; Calixto, 2015). A similar scenario could be induced by the protective microbiota.

### Calcium contribution

Active maintenance of calcium levels is required to promote regeneration, which directly involves the mitochondrial calcium uniporter *mcu-1* and the SERCA pump *sca-1* (Sarasija et al., 2018; Wang et al., 2019; Calvo-Rodriguez et al., 2020; Calvo-Rodriguez and Bacskai, 2021). The blockade of *mcu-1* prevents cellular neuronal death in the context of Alzheimer’s disease (Sarasija et al., 2018; Calvo-Rodriguez and Bacskai, 2021). Here we show that during development the silencing of both *mcu-1* and *sca-1* is required for a reduction in wild-type axons TRN autonomously, suggesting that they are redundant. It is also possible that an increase in intracellular calcium by itself is not damaging under a protective diet. Intraorganellar stress has been shown to trigger degeneration (Sarasija et al., 2018; Wang et al., 2019).

In diapausing animals, the silencing of *mcu-1* or *sca-1* halted the regeneration of TRNs. Our results contrast with the simple idea that calcium by itself only damages the cell. Silencing of *sca-1* and *mcu-1* induces higher cytoplasmic calcium concentration, but only regeneration is impaired, and an increment in neuronal death is not observed when calcium is present in the environment of diapausing animals. Earlier it was proposed that calcium toxicity may be related to an increment in mitochondrial calcium, which promotes oxidant-induced mitochondrial loss of function, ATP depletion, and mitochondrial bursting (Harman and Maxwell, 1995; Calvo-Rodriguez et al., 2020).

The reduction of intracellular calcium transporters does not simply generate an increment of calcium concentrations inside the neuron, but rather alters the subcellular pattern of calcium levels that also prevents mitochondrial bursting (Sarasija et al., 2018; Calvo-Rodriguez et al., 2020; Calvo-Rodriguez and Bacskai, 2021), suggesting that mitochondrial or ER damage is more relevant for the outcome of the cell than the cytoplasmic concentration of calcium.

Regeneration actively requires calcium (Khachaturian, 1989; Chierzi et al., 2005; Ghosh-Roy et al., 2010; Sun et al., 2014). In *E. coli* HT115 calcium depletion impaired the protection and regrowth of axons, as is observed in dauer-induced regeneration. This suggests that calcium may be crucial during axonal maintenance generally.

### Mitochondria and neuroprotection

The role of mitochondrial function in neuronal protection has been linked to the microbiota–metabolites–brain axis (Saint-Georges-Chaumet and Edeas, 2016; Nurrahma et al., 2021). The mitochondria of animals that enter diapause become larger and more numerous in *mec-4d* animals under prodegenerative pressure. Since energy production is associated with mitochondrial fusion (Skulachev, 2001), longer mitochondria may be key to the correlations found in our work and previous reports (Chen et al., 2007; Knott et al., 2008; Chen and Chan, 2010; Chang et al., 2019; Wang et al., 2019).

Intermittent fasting improves cognitive traits and mitochondrial function (Liu et al., 2020). Dauers have reduced metabolic rates and elevated levels of heat shock proteins, are resistant to oxidative stress, and exhibit a low metabolic rate compared with other larval stages (Anderson, 1982; Burnell, 1989; O’Riordan and Burnell, 1990; Dalley and Golomb, 1992; Penkov et al., 2020). Most of these characteristics are associated with increased mitochondrial functions, such as more ATP availability or a reduction of toxicity (Fariss et al., 2005; Labbadia et al., 2017). *mec-4d* dauers have longer and more mitochondria, suggesting that these traits help maintain axons (Knott et al., 2008; Chang et al., 2019). Diapause induction affects mitochondrial physiology (Artal-Sanz and Tavernarakis, 2009; Lourenço et al., 2015; Lourenço and Artal-Sanz, 2021), suggesting that the regeneration of dauers might be a consequence of an increased buffering capacity and improved energy management by larger mitochondria.

*drp-1* or *eat-3* silencing and consequently impaired mitochondrial fission/fusion did not affect neuroprotection by diet, consistent with our observation that animals fed with *E. coli* HT115 do not increase the number or size of mitochondria. However, the loss of *fzo-1*/*mitofusin-1*, which initiates the fusion of the external mitochondrial membrane that caused a dramatic reduction in AxW morphology, suggesting that mitochondrial structure influences the regenerative processes. Unsurprisingly, the silencing of genes required for mitochondrial metabolic function *cts-1* and *icl-1* affect neuroprotection in a TRN-autonomous manner, which directly impacts ATP production of the cells and their capacity to respond to stress (Zampese et al., 2022). This supports the idea that changes in the metabolism induced by the microbiota can cause the neuronal protection to be independent of an increase in mitochondrial number or size.

## Synthesis

Reviewing Editor: Karl Herrup, University of Pittsburgh School of Medicine Decisions are customarily a result of the Reviewing Editor and the peer reviewers coming together and discussing their recommendations until a consensus is reached. When revisions are invited, a fact-based synthesis statement explaining their decision and outlining what is needed to prepare a revision will be listed below. The following reviewer(s) agreed to reveal their identity: Hei-Man Chow, Danielle Mor. Note: If this manuscript was transferred from JNeurosci and a decision was made to accept the manuscript without peer review, a brief statement to this effect will instead be what is listed below.

Synthesis for eN-TNWR-0424-22X

Microbiota and diapause formation confer neuroprotection in C. elegans by different mechanisms.

The manuscript utilizes a well-established C.elegans neuronal degeneration model to investigate potential common neuroprotective mechanisms induced either by the gut microbiota or by hunger-induced diapause. The authors combine transcriptomics, genetics and cell-based results. They find that extracellular calcium is needed for neuroprotection induced by lactate-producing bacteria and diapause entry. The work represents an interesting investigation into an important question: whether different neuroprotective paradigms (beneficial diet versus starvation) act through similar mechanisms. While the results offer insights into potential points of convergence and divergence, the study does not clearly disentangle the mechanisms due to the lack of critical controls. As a result, the conclusions regarding the role of calcium in diapause, and whether a beneficial diet and diapause together have additive effects, are not well substantiated. Thus, while the results may be useful for future studies or therapeutic development, there are several important concerns that need to be addressed.

The study attempts to identify shared versus distinct mechanisms for the two neuroprotective paradigms, yet the stated conclusions are contradictory. For example, the authors state that “calcium is required under both microbiota- and dauer-induced protection” but also that these “processes occur in diverse ways, requiring differential changes in mitochondrial function and calcium homeostasis.” Even the title of the manuscript emphasizes “different mechanisms”. The authors should be clear regarding which mechanisms are found to overlap, and which are unique.

A major concern with this study is the presentation of combined neuroprotective paradigms (HT115 diet + dauer diapause), but controls showing the effects of the individual interventions alone are not reported. Much of the calcium data is presented this way, which makes the combination of HT115 and diapause difficult to interpret. This serves to undermine the authors’ ability to make conclusions on the mechanisms of diapause alone, or whether these two interventions are additive in their neuroprotective effects.

The criteria used for scoring need to be spelled out in more detail since not all readers are familiar with C. elegans cell biology or neuroanatomy. For example, the authors describe the scoring of neuronal integrity (Line 192-200). Labelled representative images of nematodes with different neuronal morphology should be provided. Similarly, how were non-fragmented, filamentous, intermediate, and fragmented mitochondria defined subjectively (Line 207-216)? How do they correlate with mitochondrial function and metabolism? Data are needed to support this argument.

More details are also needed in the criteria used for the molecular analysis. In Line 239, was the cutoff defined as log2|FC| > 1 or log10|FC| > 1? The use of p-value in DEG analysis over adjusted p must be justified. The meaning of candidate genes is unclear (Line 299-303). Did the authors perform RNAi on all 40 upregulated genes? Were the expressions of the neuroprotective candidate genes specific to TPNs (Line 303-310)? Are the differentially expressed genes in Fig 1B or C significantly enriched for any functional characterizations?

The authors have investigated the effects of the strain of E coli used to feed nematodes growing in calcium-free environments. There is some concern that the neuroprotective effects may be due to the supplementation of intracellular calcium from the bacteria themselves. Do HT115 have a higher intracellular calcium content than OP50? Measurements of the bacterial calcium levels would address this concern. Why do the authors think that calcium chelation shows opposite effects in OP50- versus HT115 mec-4d animals in Fig 2A and B?

The authors claim that “energy availability is a requirement for axonal regeneration and maintenance” (Line 424-427) based solely on the observed mitochondrial morphology and the silencing citrate synthase. To support this argument better, the authors should directly quantify energy production/availability in TRNs (e.g., by measuring ATP levels and mitochondrial membrane potentials). It would also be interesting to know whether HT115 can rescue the energetic deficits. Do the authors believe that these factors act in a signaling cascade from intestine to neurons? What do the authors make of the specific genes identified in Table 1, in terms of how they may coordinate cross-tissue signaling and neuronal viability?

For the transcriptomic analysis (Figure 1), it would improve the work if the authors could provide a PCA plot to show that different groups are distinct. Other suggestions would be to include a p-value histogram to show that the statistical analysis is sufficiently powered, and possibly a volcano plot. The authors should clarify whether the functional enrichment conducted on common DEGs in systemically and TRN-specific RNAi worms? The authors should specify the list of genes used. Is there any difference in functional characterization of systemically-required versus TGN-specific genes validated by RNAi in Fig 1? It appears that Fig 1G does not distinguish between these groups. Also, it is not clear if Fig 1G or Fig S1 enrichment scores are statistically significant.

In figure Figure 3A it appears that WT worms have shorter mitochondria that occur at lower density. This contradicts the quantifications in Figure 3C-G. Higher magnification insets of both WT and mec-4d mitochondria should be provided. To support the quantification in Figure 3C-F, representative images of mitochondrial morphology should be provided. While bar-graph quantifications of the axonal phenotype in mec-4d worms are important, it is necessary to show representative images of axons alongside the quantifications throughout the manuscript.

The authors state 4 genes are upregulated in dauers, but then only name 3 genes (Lines 313-314). Also, some explanation is needed for why downregulated genes were not included.

On page 16 lines 339-341, the authors state “At day 14 post diapause, AxW further increased in HT115 controls, suggesting that diapause can induce additional neuroprotection on top of diet-induced benefits.” Again, this conclusion is unsubstantiated. To make this claim, the authors must compare HT115 dauers to HT115 non-dauers. However, only dauers are shown.

The interpretation of Fig 2E-F as “both transporters are required for diet-induced neuroprotection” is incorrect. Instead, the data show that at least one of these transporters must be present for diet-induced neuroprotection.

The Discussion section feels very informal in the style that it is written. It also at times feels more like a Results section, focusing on very granular details of the data. The end of the Discussion especially if it were written more broadly and tied the whole study together.

Minor comments:

This sentence (Line 496-499) needs to be clarified: “If intracellular calcium transporters are available, this is, the increase of calcium in the cytoplasm is not the starting point, it could be possible that intra-organelle stress is the trigger for degeneration.”

Figure 1B-C: The legend of the color scale is missing.

In Fig 1C, do the authors have an explanation for why a group of genes are selectively downregulated at 24 hours?

The authors state that 40 genes were upregulated in Fig 1B, yet only 29 are tested by RNAi in Fig 1C-D. Why were the remaining 11 genes excluded?

Figure 1D-E, Figure S1A-F: It is not clear what the meaning is. The same genes are represented by different colors in different figure panels, and those colors are also reused for functional terms, which is quite confusing.

In Table 1, some genes required in touch receptor neurons do not have neuronal expression listed. How do the authors explain this?

It would improve clarity if the y-axes in the various panels of Fig 2 were the same.
