# Supplementary figures and images for: Microbiota and Diapause-Induced Neuroprotection Share a Dependency on Calcium But Differ in Their Effects on Mitochondrial Morphology
Source: eNeuro. 2023 Jul 21;10(7):ENEURO.0424-22.2023. doi: 10.1523/ENEURO.0424-22.2023 (PMC10368204; doi:10.1523/ENEURO.0424-22.2023)

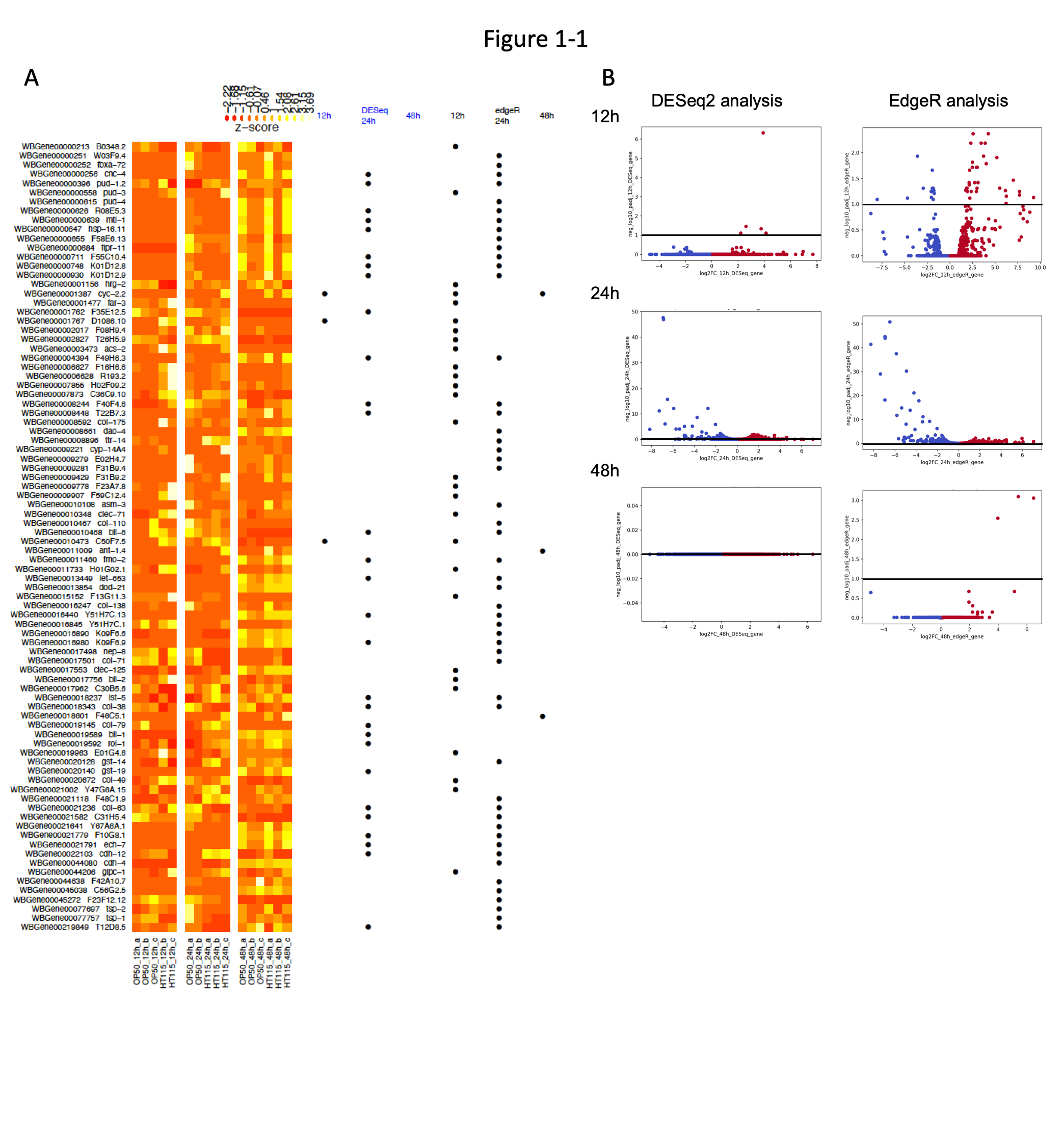

Supplement: Figure 1-1 — A, Heatmap of genes DE in E. coli HT115 compared with E. coli OP50 at different times during development. DeSeq and EdgeR analyses are shown. B, Volcano plot for DE analysis on each developmental timepoint. Download Figure 1-1, TIF file. [file enu-eN-NWR-0424-22-s01.tif]

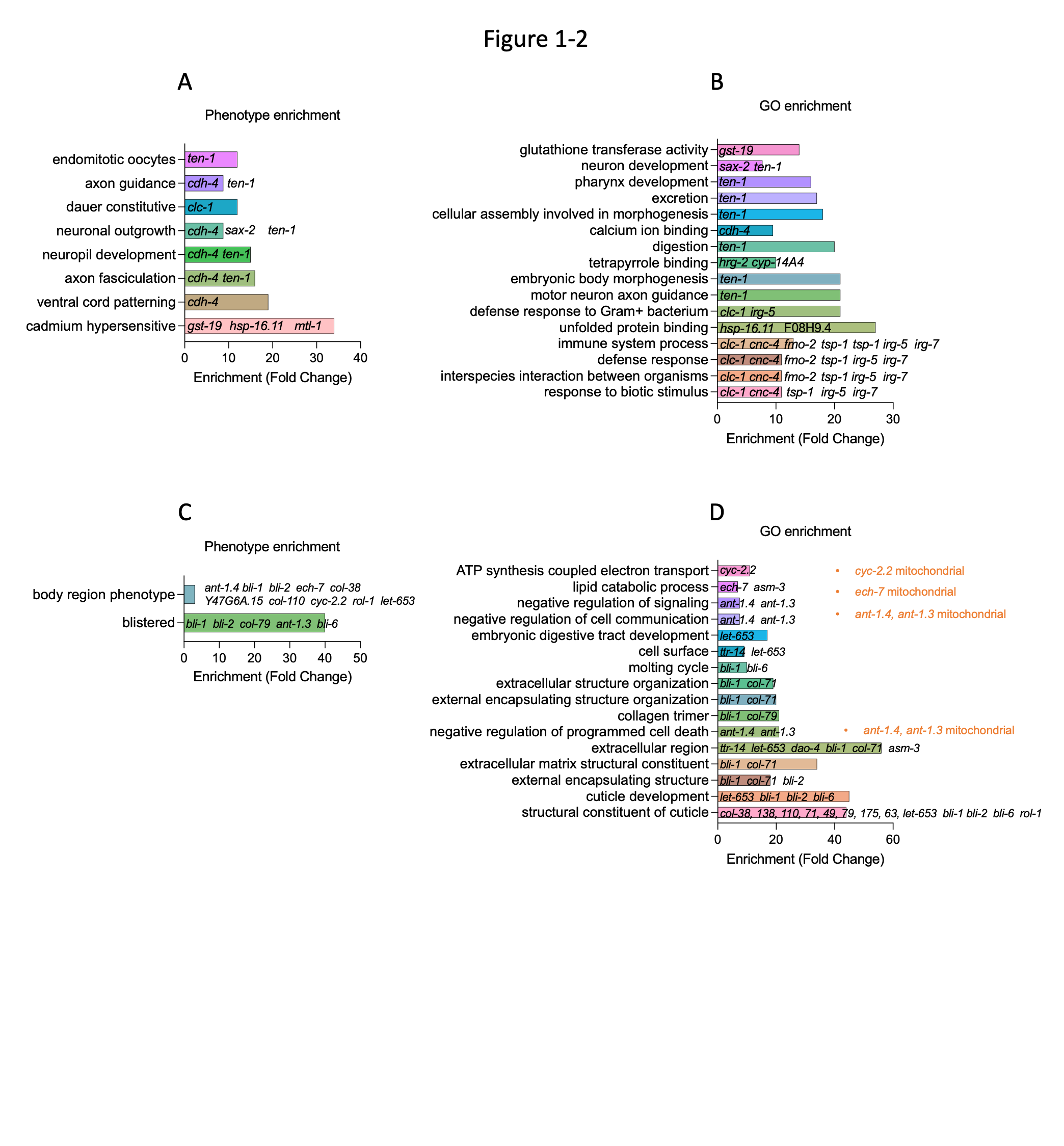

Supplement: Figure 1-2 — Enrichment analysis of genes differentially expressed in E. coli HT115 diet. A–D, Phenotype (A, C) and gene ontology (B, D) enrichment of genes upregulated (A, B) and downregulated (C, D) in E. coli HT115 compared with E. coli OP50. Statistical analyses are shown in Extended Data Table 1-3. Download Figure 1-2, TIF file. [file enu-eN-NWR-0424-22-s02.tif]

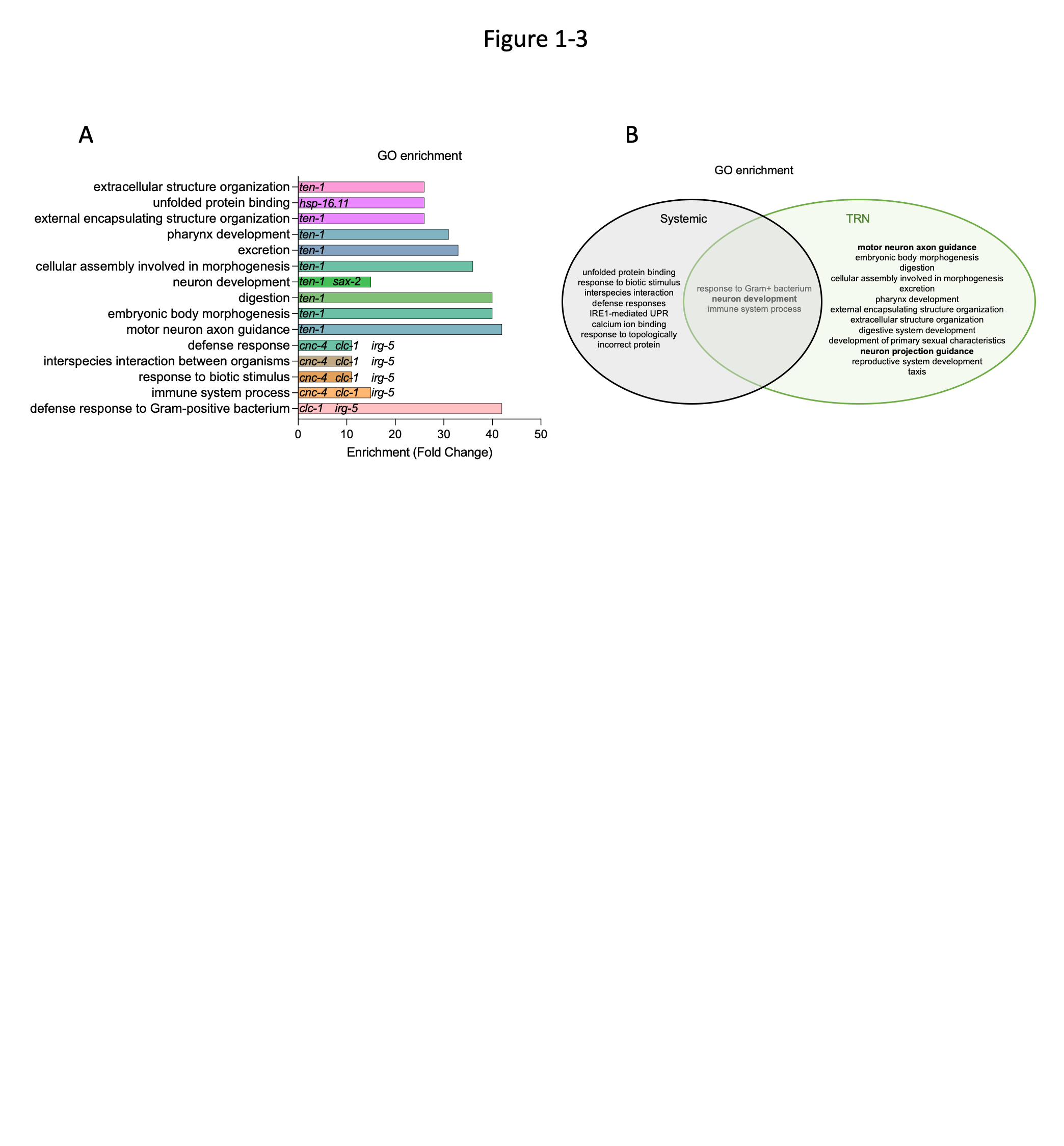

Supplement: Figure 1-3 — Enrichment analysis of genes required for neuroprotection conferred by E. coli HT115. A, Gene ontology of genes that are required for E. coli HT115 neuroprotection. B, Venn diagram of Gene ontology categories of genes required systemically and in the TRNs for neuroprotection. Statistical analyses are shown in Extended Data Table 1-3. Download Figure 1-3, TIF file. [file enu-eN-NWR-0424-22-s03.tif]

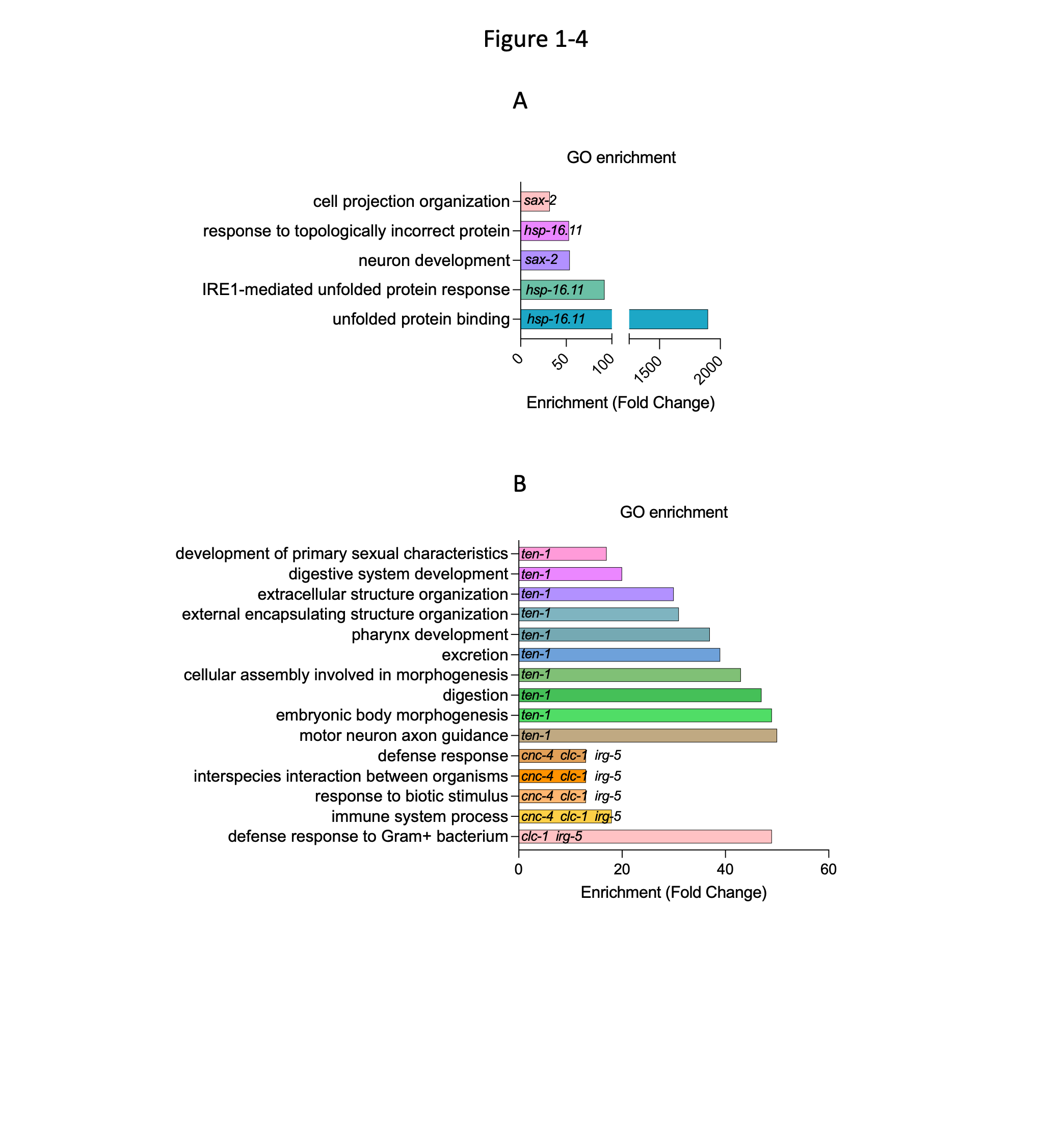

Supplement: Figure 1-4 — A, B, Enrichment analysis of genes upregulated in dauers that are shared with genes required for neuroprotection in E. coli HT115. A, B, Gene ontology enrichment associated with genes shared between E. coli HT115 RNAi-positive clones and dauers (A), and those not shared with dauers (B). Statistical analyses are shown in Extended Data Table 1-3. Download Figure 1-4, TIF file. [file enu-eN-NWR-0424-22-s04.tif]
